# Scoping Review of International Experience of a Dedicated Fund to Support Patient Access to Cancer Drugs: Policy Implications for Thailand

**DOI:** 10.34172/ijhpm.2023.7768

**Published:** 2024-01-28

**Authors:** Parnnaphat Luksameesate, Osot Nerapusee, Chanthawat Patikorn, Puree Anantachoti

**Affiliations:** Department of Social and Administrative Pharmacy, Faculty of Pharmaceutical Sciences, Chulalongkorn University, Bangkok, Thailand

**Keywords:** Dedicated Fund, High-Cost Drug, Cancer Drug, Global Review, Thailand

## Abstract

**Background:** Access to high-cost cancer drugs is an unsolved problem globally. The dedicated drugs fund is attractive and feasible. This study reviewed currently implemented dedicated drugs fund worldwide to inform policy implications for Thailand.

**Methods:** A scoping review was conducted to identify countries currently implementing dedicated funds for cancer drugs. We searched electronic databases, PubMed and Embase, from 2010 to May 2021, Google and Google Scholar in August 2021, and government websites up to April 2022. The structure, management, cost containment strategies, and impact of dedicated funds were summarized and compared across the identified countries and Thailand.

**Results:** Out of 218 nations, Hong Kong, England, and Italy have established dedicated cancer drugs fund, primarily funded by their governments. Funds in England and Italy operate within annual budget limits. Hong Kong relies on an endowment fund. In England and Italy, pharmaceutical companies contribute proportionally to cover overspending as per risk-sharing agreements, while cost-sharing is not required. Hong Kong implements cost-sharing based on a patient's family income. England and Italy employ a parallel pathway, utilizing the same drug selection committee to determine whether innovative drugs belong in the regular pharmaceutical benefits package or the dedicated drugs fund. Hong Kong follows a sequential pathway, allowing drugs to be considered for the dedicated funds after a negative decision. These countries use the fund for 5-11 years, making administrative adjustments to ensure sustainability.

**Conclusion:** The dedicated drugs fund is an effective strategy to improve access to non-reimbursable high-cost drugs in Thailand. Robust evaluation of the fund itself and funded drugs are recommended for policymakers’ better decision-making. Learning from other countries can offer promising solutions. Health insurers need to balance providing cancer treatments with overall system preparedness.

## Background

 Cancer is a group of diseases with uncontrolled cell growth. Cancer can occur in many organs. GLOBOCAN revealed 19.3 million new cancer cases from 36 cancer types worldwide in 2020.^1^ According to the 2019 World Health Organization (WHO) report, cancer was ranked second among the leading causes of death worldwide.^2^ In Thailand, cancer is the leading cause of mortality, with a reported rate of 112.8-125.0 per 100 000 people from 2015 to 2019.^3^ Global expenditure on cancer treatment is tremendous, and drug cost consumes the largest proportion of money in terms of medical treatment. Between 1995 and 2018, health expenditures for cancer treatments in European Union countries nearly doubled from US$ 61 billion (52 billion Euro) to US$ 121 billion (103 billion Euro). During the same period, there was an increase in newly diagnosed cancer cases by approximately 50% and the use of high-cost drugs. Therefore, it was expected that the costs would continue to increase.^4^

 To improve access to cancer drugs, various strategies have been utilized. Basic pharmaceutical reimbursement schemes with pricing policies provide the basic tools for most health insurance systems. Alternative funding strategies specific to cancer drugs, such as managed entry agreements (MEAs), dedicated funds for cancer drugs, orphan drug reimbursement policy, adjusted cost-effectiveness threshold, and the use of compulsory licensing, were complementarily utilized on top of the preferred drug list strategy. Financial assistance is another strategy used by either government or non-government organizations to support patients. Examples of financial assistance strategies include providing additional health insurance policies for the poor, reducing or exempting patient cost-sharing, utilizing patient assistance programs (PAPs), and setting up assistance foundations. Combinations of these strategies have been utilized and implemented to fit each country’s circumstances.^5^

 Thailand’s health insurance system is highly recognized by the international community as a strong and advanced system comparable to the health insurance systems of other high-income countries. In Thailand, health insurance schemes cover all Thai citizens; Social Security Scheme (SSS) for employees in the private sector; Civil ServantMedical Benefit Scheme (CSMBS) for government officers and their dependents; and universal health coverage (UHC) schemes for the rest.

 In general, access to medical services for Thais is considered good. All Thais under insurance have access to drugs listed in the National List of Essential Medicines (NLEM) of Thailand. However, access to cancer drugs has been reported to be quite limited, and there is a huge gap between local cancer protocol and international cancer treatment guidelines.^6^ Saerekul et al in 2018 and Patikorn et al in 2019 unanimously found that more than 85% of approved cancer drugs worldwide were market-authorized by the Thai Food and Drug Administration; however, only half of them were reimbursable.^6,7^ Patikorn et al also found that access to cancer drugs differed across the three Thai public health insurance schemes. SSS provides fewer types of cancer coverage when compared to UHC and CSMBS. CSMBS has broader coverage as they have an additional Oncology prior authorization (OCPA) program that allows for better access to cancer drug items and indications not listed in the NLEM. OCPA was established in 2005 with six cancer drugs. After more than 10 years of hiatus, OCPA has resumed its activity and actively included more cancer drugs in its program since 2018. Furthermore, there is a concern regarding the local and international cancer treatment guidelines. An example of treatment recommendations for advanced or metastatic non-small cell lung cancer with anaplastic lymphoma kinase rearrangement positive is the use of anaplastic lymphoma kinase inhibitor drugs, as recommended by the National Comprehensive Cancer Network guideline 2021.^8^ However, the local cancer reimbursement protocol 2015 in Thailand recommends platinum (cisplatin or carboplatin) with paclitaxel, vinorelbine, docetaxel, gemcitabine or pemetrexed instead.^9^

 Thailand has a health technology assessment (HTA) body supporting drug reimbursement decisions. Thai NLEM is updated yearly and implemented across the three public health insurance schemes. Reimbursement of cancer drugs must follow the cancer protocols.^6^ Reimbursement decisions have been primarily limited by the affordability of the health system, such as the incremental cost-effectiveness ratio (ICER) threshold in Thailand. The results of three cost-effectiveness analyses of high-cost innovative cancer drugs conducted by the Health Intervention and Technology Assessment Program (HITAP) in 2017 showed that the ICER per quality-adjusted life year (QALY) gained ranged from US$ 20 099 to US$ 353 842 (682 155-12 009 328 Thai Baht) which far exceeded Thailand’s cost-effectiveness threshold of US$ 4714 (160 000 Thai Baht) per QALY gained by 4-75 times.^10-12^ Therefore, we have still encountered the access problem of high-cost cancer drugs due to the very high ICER/QALYs gained. Moreover, consideration of generic or biosimilar availability is an issue. To illustrate, the NLEM includes five monoclonal antibodies (rituximab, trastuzumab, basiliximab, tocilizumab, and bevacizumab) and four targeted therapies (erlotinib, imatinib, nilotinib, and dasatinib). More importantly, some of these drugs were listed in the NLEM at the time when generic products were available.^6^ This implies that patients may experience delays in accessing care. In addition, the policy of choosing only one drug from the same pharmacological drug class was utilized.^13^ Thus, many cancer drugs in Thailand are not reimbursable, and these drugs compose not only non-innovative but also the innovative drugs.

 Besides the preferred drug list, pricing policy, prior authorization, economic evaluation, and budget impact strategies implemented in Thailand’s public health insurance systems, the PAP is another financing strategy.^6^ Several pharmaceutical companies sponsor PAPs, which cover a range of cancer drugs to alleviate self-paying patients financially.^14^ There are many types of PAPs; however, they could be broadly categorized into two groups.^6,14^ First is the fixed scheme in which PAP offers a fixed assistance pattern of “Buy X gets Y boxes free” for every patient under the same indication. The second is PAP which considers patients’ income. Income is a major criterion in deciding whether the patient is qualified for PAP or not and reflects the level of financial support the patient will receive. The decision to join PAP depends solely on the patient. Although PAP could alleviate the financial burden of the patients, it helps only those who still have the ability to pay and leaves out the poor who cannot afford it.^6,14^

 Thailand has utilized many strategies to enhance access to cancer drugs. However, dedicated funds for cancer drugs have not been explicitly implemented. Dedicated funds are another financial subsidization method that some countries use to aid access to high-cost cancer drugs. National budgets are allocated to subsidize cancer drugs awaiting reimbursement decisions.^15,16^ Among other strategies not yet implemented in Thailand, the dedicated fund is one of the interesting strategies that could benefit our current healthcare system. As countries which have implemented dedicated funds have different criteria and processes, it is interesting to explore the details of those dedicated funds. Therefore, this study aims to review existing international dedicated funds for cancer drugs to inform the development of policy implications suitable for Thailand.

## Methods

###  Data Source

 We conducted a scoping review. Because of the nature of the studies on policies and routine operations, information related to cancer drug funds is not solely available in peer-reviewed journals. A literature review of published literature alone may miss out on important information. Therefore, we searched for both published literature and grey literature to identify countries that currently implement dedicated funds for cancer drugs. We reported following the Preferred Reporting Items for Systematic Reviews and Meta-Analyses extension for Scoping Reviews (PRISMA-ScR) checklist (Supplementary file 1 – Table S1).^17^

 For the purpose of this study, dedicated funds for cancer drugs were defined as special reimbursement programs with the allocated budget to provide patients access to cancer drugs outside the regular reimbursement system (ie, reimbursement list) awaiting to be transitioned to the standard reimbursement drugs list. A case-by-case reimbursement of cancer drugs was not included under this definition. In addition, dedicated funds that ceased their operation were not included.

 Electronic databases, including PubMed and Embase, were searched to identify countries implementing dedicated funds for cancer drugs from any relevant peer-reviewed articles published from 2010 to May 12, 2021. This is to capture the latest ten years of evidence using a combination of synonyms of “Cancer,” “Drug,” and “Fund.” Search strategies are shown in Supplementary file 2 – Table S2. Titles and abstracts of the identified articles from electronic databases were then independently screened for relevancy by two reviewers (PL and CP) after excluding duplicates. Relevant articles were then sought to retrieve their full-text articles and independently selected against the eligibility criteria by two reviewers (PL and CP). The third reviewer (ON) made a final decision when a discrepancy was identified. Eligible articles should be full-text articles, including original research, reviews, and editorials published in peer-reviewed journals which provide information regarding the dedicated funds for cancer drugs. Any discrepancies in this review were resolved by discussion among all authors.

 Grey literature, including websites and reports, were searched via Google and Google Scholar in August 2021 using the keywords “Cancer” AND “Drug” AND “Fund” AND “Name of country” for additional information regarding the dedicated funds for cancer drugs in 218 countries throughout the world. Only the first 50 articles identified from Google and Google Scholar searches were screened. We then further searched the government websites to gather information on dedicated funds for cancer drugs. Searching the government websites was performed up to April 30, 2022.

 Selected evidence was independently extracted by two reviewers (PL and CP). The third reviewer (PA) made a final decision when a discrepancy was identified. The following information was extracted: country, name of dedicated funds for cancer drugs, year of establishment, responsible organization, organization structure, mission and objective of dedicated funds, financing mechanism, selection criteria and coverage, operational process, cancer drugs list, and impact of dedicated funds for cancer drugs on patient access.

###  Data Analysis

 Extracted data were analyzed to compare the structure of dedicated funds for cancer drugs, management, and cost containment strategies, and the impact of the dedicated fund on patient access across countries. Cancer drugs under the dedicated funds were extracted and compared across the identified countries and Thailand to understand the current situation of access to cancer drugs in Thailand. Findings and extracted data were subsequently used to formulate policy recommendations for dedicated funds for cancer drugs in the context of the health system in Thailand.

 To analyze the current situation of access to cancer drugs in Thailand, the cancer drug items of each country were compared to their reimbursable classification. Identified cancer drugs were divided into 15 groups according to the Anatomical Therapeutic Chemical (ATC) classification system.^18^ The reimbursable classification of cancer drugs was divided into five categories as follows: (1) Reimbursable under regular benefits package: drug could be reimbursed through the national health insurance scheme in the country; (2) Reimbursable under special programs: drug could be reimbursed through the special programs outside the regular benefits package including OCPA in Thailand; (3) Reimbursable under the dedicated funds: drug could be reimbursed through the dedicated funds in the identified countries; (4) Not Reimbursable: drug could be neither reimbursed through the national health insurance scheme nor the special program in the country; and (5) Not registered: data were not registered, or the drug had not been approved for marketing authorization in the country. For Thailand, we reported access to cancer drugs separately as UHC/SSS and CSMBS due to the differences in access to cancer drugs across these three health insurance schemes.

## Results

###  Identified Countries With Dedicated Funds for Cancer Drugs

**Figure 1:**
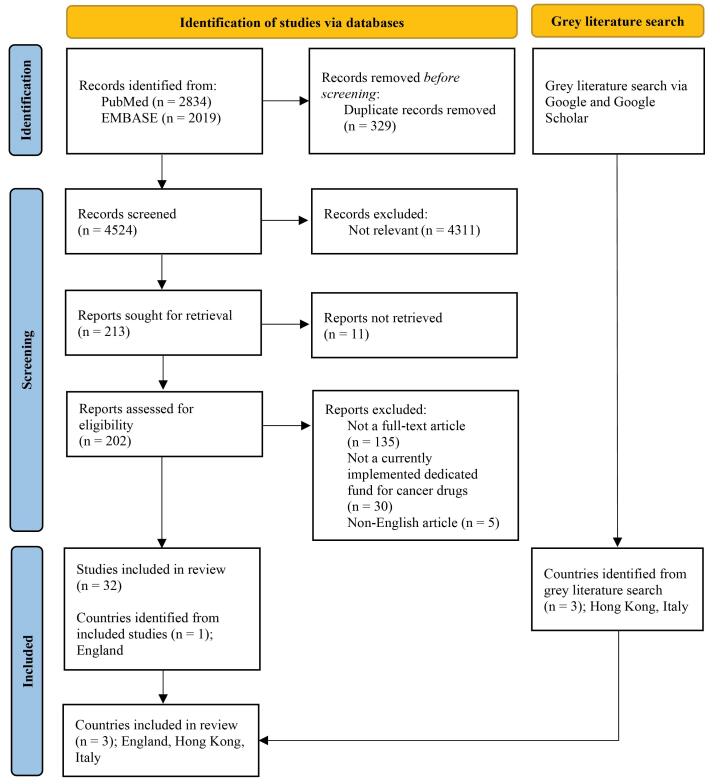
Selection Flow of Countries That Currently Implement Dedicated Funds for Cancer Drugs.

 A total of 4853 articles were identified from electronic databases, of which 33 were included (Figure 1).^16,19-49^ From these included articles, dedicated funds for cancer drugs were found to be currently implemented in England. A supplemental search for dedicated funds for cancer drugs in 218 countries via Google and Google Scholar found three countries: Hong Kong, England, and Italy. We found no countries that had previously implemented dedicated funds programs for cancer drugs and ceased their operations.

 Country demographics and health insurance systems of these three countries and Thailand were summarized in Table 1. Details of dedicated funds for cancer drugs in England, Hong Kong, and Italy were summarized in Table 2. We compared key characteristics of dedicated funds for cancer drugs in Hong Kong, England, and Italy.^37,43,50-61^ Dedicated funds for cancer drugs in England and Italy are similar in providing early access to innovative cancer drugs while real-world data are being collected to inform future transitioning to the normal funding mechanism. On the other hand, dedicated funds for cancer drugs in Hong Kong have been established to provide financial assistance to patients who need cancer drugs and are transitioning to reimbursement. England’s cancer drugs fund (CDF) allows access to only cancer drugs. Hong Kong’s Samaritan Fund (SF) and community care fund (CCF) and Italy’s Fund for Innovative Oncological and Non-oncological Medicines provide access to both cancer and non-cancer drugs.

**Table 1:** Country Demographics and Health Insurance System as of 2020

	**Thailand**	**Hong Kong**	**United Kingdom**	**Italy**
Population, million	69.80	7.50	67.20	59.60
GDP, million USD	501 644	346 585	2 707 743	1 886 445
GDP per capita, USD	7187	46 324	40 285	31 676
Total healthcare expenditure per capita, USD	296	3250	5268	3819
Healthcare expenditure, % of GDP	3.8%^a^	6.1%	9.8%	8.9%
Pharmaceutical expenditure, % of total healthcare spending	55.5%^b^	10.6%	11.5%^a^	18.0%^a^
Public healthcare spending per capita, USD (% of total healthcare expenditure)	216 (73.0%)	1740 (53.5%)	4306 (81.7%)	2914 (76.3%)
National health insurance system	NHSO	Public Healthcare System	NHS	SSN
Healthcare coverage scheme	- UHC - SSS - CSMBS	- UHC - CSSA	UHC	UHC
Health benefits packages	- Healthcare services: outpatient, inpatient, health promotion and disease prevention, high-cost healthcare services - HIV infection care - Chronic kidney disease care - Prevention of chronic diseases in community-based care - Long-term care for dependent patients - Primary care cluster	- Pharmaceuticals - Inpatient care - Preventive medicine - Outpatient specialist care - Maternity care - Home care - Primary care - Hospice care	- Pharmaceuticals - Inpatient and outpatient hospital care - Preventive services - Maternity care - Physician services - Clinically necessary dental care - Some eyes care - Mental healthcare - Palliative care - Some long-term care - Rehabilitation - Home visits by community-based nurses - Wheelchairs, hearing aids, and other assistive devices	- Pharmaceuticals - Inpatient care - Preventive medicine - Outpatient specialist care - Maternity care - Home care - Primary care - Hospice care
Classifications of pharmaceutical benefits	- List A: Standard medicines for preventing and treating common health problems - List B: Alternative medicines to List A medicines - List C: Medicines prescribed in specialty diseases - List D: Medicines with many indications that are likely to be misused - List E1: Medicines for special programs proposed and responsible by government organizations - List E2: Very high-cost medicines for specific groups of patients	- General drug - Special drug - Self-financed items drug with Safety Net - Self-financed items drug without Safety Net	- Prescription-only medicine - Pharmacy - General sales list - Black-listed - Selected list	- Class A: lifesaving drugs and treatments for chronic conditions - Class H: drugs delivered only in a hospital setting - Class C: non-reimbursable drugs - Class C (non negotiated): drugs identified as innovative status covered by Fund for innovative oncological and non-oncological medicines
Cost sharing of pharmaceutical benefits	None	- General drugs & special drugs: full coverage by public healthcare system - Self-financed drugs with safety net cost sharing by patients <20% depending on household income - Self-financed drugs without safety net: 100% out of pocket	Copayment of US$ 12.50 per outpatient prescription - People who are exempt from prescription drug copayments include: - Children aged 15 and under - Full-time students aged 16 to 18 - People aged 60 or older - People with low incomes - Pregnant women and women who have given birth in the past 12 months - People with cancer and certain other long-term conditions or disabilities	Reimbursable - Tier 1 (Class A): prescription fee for several regions - Tier 3 (Class H): no cost sharing Non-reimbursable - Tier 2 (Class C): 100% out of pocket - Class C (non negotiated): no cost sharing
Reimbursement decision making system	Centralized decision making	Centralized decision making	Decentralized decision making across England, Scotland, Wales, and Northern Ireland	Decentralized decision making
HTA body	HITAP	DAC	NICE	AIFA
References	^62-64^	^50,62,65,66^	^62,65,67,68^	^62,65,69,70^

Abbreviations: AIFA, Agenzia Italiana del farmaco (Italian Medicines Agency); CSMBS, Civil Servant Medical Benefit Scheme; CSSA, Comprehensive Social Security Assistance; DAC, Drug Advisory Committee; GDP, gross domestic product; HITAP, Health Intervention and Technology Assessment Program; NHS, National Health Service; NHSO, National Health Security Office; SSN, Servizio sanitario nazionale (Italian National Health Service); SSS, Social Security Scheme; NICE, National Institute for Health and Care Excellence; UHC, universal health coverage; USD, United States dollar; HTA, Health technology assessment. Costs are presented in 2020 USD to ease comparison. ^a^ Data as of 2019. ^b^ Data as of 2014.

**Table 2:** Characteristics of Dedicated Funds for Cancer Drugs in Hong Kong, England, and Italy

	**Hong Kong**		**England**	**Italy**
Name of a dedicated fund for cancer drugs	SF	CCF Medical Assistance Program	CDF England	Fund for Innovative Oncological and Non-oncological medicines
Establishment year	1950	2011	2010 (reformed in 2016)	2017
Objectives	Financial assistance for self-financed drugs and medical devices when patients have a clinical need with access difficulty	Financial assistance for cancer drugs, uncommon disorders and medical devices not covered by the SF before transitioning to SF	Early access to cancer drugs before transitioning to the normal funding mechanism	- Early access to innovative drugs before transitioning to normal funding mechanism - Ensure equity of access to innovative drugs across regions
Administrative organization	Samaritan fund management committee	CCF administrative committee	NHS England	SSN
Responsible sector	Government and non-government	Government	Government	Government
Source of funding	- Government grants - Social Welfare Department - Donations	- Government - Donations	NHS England	Central government
Annual budget allocated, million USD (% of pharmaceutical expenditure)	2012: 1300 for 2012 to 2022 (NA)		- 2011-2015: 256 (NA) - 2016: 436 (1.1%)	1136 which could be crossed paid between oncology and non-oncology drugs (2.4%) - 568 for oncological drugs (1.2%) - 568 for non-oncological drugs (1.2%)
An annual budget used, million USD	- 2011: 27 - 2012: 31 - 2013: 38 - 2014: 43 - 2015: 47 - 2016: 50 - 2017: 55 - 2018: 54 - 2019: 68 - 2020: 88 - 2021: 110	2017: 22 2018: 36 2019: 40	- 2013: 359 - 2014: 436 - 2015/2016: 598 - 2017/2018: 259 - 2018/2019: 308 - 2019/2020: 407 - 2020/2021: 431	- 2017: 764 - 2018: 1546 - 2019: 1965 - 2020: 2279
Financial administrative organization	Samaritan fund committee	CCF medical assistance program task force	Joint NHS England/NICE/CDF Investment Group	SSN
HTA organization	Drug advisory committee	Drug advisory committee	NICE England	AIFA
Drug indications covered	Cancer and non-cancer	Cancer and non-cancer	Only cancer	Cancer and non-cancer
Coverage scheme	Self-financed items drug with the safety net	Self-financed items drug with the safety net	Conditional reimbursement with further evidence collected to address clinical uncertainties	Conditional reimbursement with further evidence collected to address clinical uncertainties
Length of coverage	Not specified (Note: for a patient who is qualified for the drug in SF/CCF, the reimbursement lasts for 18 months after which the patient’s household income will be re-evaluated)	Not specified (Note: for a patient who is qualified for the drug in SF/CCF, the reimbursement lasts for 18 months after which the patient’s household income will be re-evaluated)	Normally up to 2 years	- Fully innovative: up to 36 months (same coverage across regions) - Conditionally innovative: up to 18 months (coverage may vary across regions)
Cost sharing	Yes	Yes	No	No
Patient qualification	Hong Kong Citizens with conditions and indications approved by SF and agree to copay	Hong Kong Citizens with conditions and indications approved by CCF and agree to copay	NHS England eligible patients with conditions and indications approved by the CDF	SSN eligible patients with conditions and indications approved by the AIFA
Monitoring and evaluation	Post-approval monitoring and audit	Post-approval monitoring and audit	CDF registry to monitor and evaluate patients’ outcomes	Registry to monitor and evaluate patients’ outcomes
Maximum number of covered patients	Not specified	Not specified	Not specified	Not specified
Financial control mechanism	Tender	Tender	Financial agreement by NHS England with pharmaceutical companies with input from NICE’s cost-effectiveness analyses	Financial agreement by SSN with pharmaceutical companies with input from AIFA’s cost-effectiveness analyses - MEAs - Confidential discounts
Financial control mechanism in the event of budget overspending	The government secured a budget allocation	The government secured a budget allocation	Proportional rebate to all pharmaceutical companies receiving any funding	Rebate to all pharmaceutical companies receiving any funding
References	^50,51^	^52,53^	^37,43,54-58^	^59-61^

Abbreviations: AIF, Agenzia Italiana del farmaco (Italian Medicines Agency); CCF, community care fund; CDF, cancer drugs fund; NA, not applicable; NHS, national health service; NICE, National Institute for Health and Care Excellence; SF, Samaritan fund; SSN, Servizio sanitario nazionale (Italian National Health Service); USD, United States dollar; HTA, Health technology assessment; MEAs, managed entry agreements. Costs are presented in 2020 USD to comparison.

 The process of enlisting cancer drugs to the dedicated funds in England and Italy is done as part of the normal reimbursement pathway. Responsible authorities make the decision to include cancer drugs either under the standard drug list or dedicated funds for cancer drugs. Cancer drugs considered for listing in Hong Kong’s SF/CCF were once rejected for inclusion in the standard drug list.

 The length of coverage of cancer drugs under the dedicated funds ranges from up to 24 months in England to 36 months in Italy, after which the covered cancer drugs will be re-evaluated to make a final decision to either be reimbursable drugs under the standard drugs list or non-reimbursable drugs. Cancer drugs under dedicated funds in England and Italy will be provided under patient registry programs to monitor patient outcomes and collect real-world data. Patient outcomes are not collected in Hong Kong’s SF/CCF. Cancer drugs under the dedicated funds are provided without cost sharing in England and Italy. Hong Kong SF/CCF requires patients to co-pay depending on their household income.

 Dedicated funds for cancer drugs in all countries are funded with the annual budget allocated mainly from government sources. The expenditures of dedicated funds for cancer drugs as a percentage of pharmaceutical expenditures were found to range from 1% in England to 5% in Hong Kong. In Hong Kong, spending of the dedicated funds for cancer drugs was well controlled, with spending within the allocated budget. Spending of the dedicated funds for cancer drugs in England and Italy were exceeding the designated budget. However, the overspending did not cost extra budget to the government. England’s and Italy’s dedicated funds for cancer drugs have a financial control mechanism to prevent budget overspending, such as a proportional rebate to all pharmaceutical companies receiving any funding from the dedicated funds in the event of an overspend in England and Italy.

###  Hong Kong – Samaritan Fund and Community Care Fund Medical Assistance Programs

 SF, established in 1950 by the Legislative Council, is a charitable fund to provide financial assistance to patients who agree to co-pay of 0%-20% self-financed products with the safety net either partially or fully, depending on their household income.^71^ For a patient qualified for the drug in SF/CCF, the reimbursement lasts for 18 months, after which the patient’s household income will be re-evaluated.^50^

 The CCF Medical Assistance Program, launched in 2011, is a safety net mechanism providing additional funding to support products that the SF does not cover. Similar to SF, a co-pay is also applied. Both funds cover costly cancer drugs, non-cancer drugs, and non-drug items. The Hospital Authority agency, under the Food and Health Bureau’s supervision, is responsible for managing the SF and CCF funds.^72^ They hold regular quarterly meetings to review and update the hospital formulary. Costly products are reviewed every six months for listing into SF and CCF. However, there is no information on how long drugs are covered under the SF and CCF. As of January 2021, 51 and 37 drug items were included in SF and CCF listing, respectively.^73,74^

 In June 2012, The Finance Committee of the Legislative Council approved a commitment fund of US$ 1.3 billion for 10 years of SF operation. For CCF operation, the government has provided the majority of funds, with approximately 5% of the contribution from the private sector since 2011.^75^ As of August 2021, the CCF’s balance reached US$ 1600 million.^72^ Drug expenditure for SF and CCF medical assistance programs was estimated to be around US$ 273 million in 2021 and US$ 403 million in 2022.^50^

###  England – Cancer Drugs Fund 

 In 2010, England initiated the CDF as a mechanism to provide faster access to expensive cancer drugs that have not been reviewed, approved, or received a negative recommendation by the National Institute for Health and Care Excellence (NICE).^16^ The annual budget allocation was originally US$ 256 million.^76^ However, the CDF significantly overspent beyond the allocated budget. The annual spending increased from US$ 359 million in 2013 to US$ 436 million in 2014 and US$ 598 million in 2015/2016.^55^ Since July 29, 2016, the system has been reformed to prevent the CDF from overspending. The critical aspect of the CDF re-organization was that the CDF utilized the NICE process for reviewing new cancer drugs. The new CDF is now a managed access scheme with clear entry and exit criteria.^76^

 Previously unreviewed or unapproved cancer drugs by NICE would be enrolled in the CDF. The CDF panel, including clinical professionals, public health representatives, and patient representatives, is responsible for appraising drugs regarding access to the CDF list. New drugs are included in the list after the Chemotherapy Clinical Reference Group has reviewed clinical evidence.^54^ Clinical data are collected on the benefit of the treatments for future reconsideration after two years of coverage.^56^ Since 2016, all new systemic cancer drug indications expected to receive marketing approval are appraised by NICE for reimbursement. NICE is allowed to make one of three recommendations: (1) recommended for routine commissioning, (2) not recommended for routine commissioning, and (3) recommended for use within the CDF.^76^

 A cancer drug recommended by NICE for use within the new CDF will be funded through the CDF which acts as a new managed access fund for resolving uncertainty. Pharmaceutical companies and the National Health Service (NHS) England will need to agree with the managed access agreement consisting of two key components: a data collection arrangement and a CDF commercial agreement.^76^

 The data collection arrangement includes the data needed to be collected to resolve significant clinical uncertainties jointly set on a case-by-case basis by NHS England, NICE, Public Health England, and the pharmaceutical company, with input from patients and clinicians.^76^ The time frame for a data collection period is determined on a case-by-case basis, depending on the level of uncertainties. The time frame is designed to be as short as possible but usually would be up to two years.^77^ However, the accurate duration of drugs would be considered on a case-by-case basis.^25^

 The CDF commercial agreement determines how much NHS England will pay during the managed access period with a confidential agreement between NHS England and the pharmaceutical company. The extent to which the drug costs are covered by NHS is determined on a case-by-case basis with input from NICE based on the results of the cost-effectiveness estimates. After the managed access period, drugs covered under CDF will be reappraised in which the decision could be (1) recommended for routine commissioning, (2) not recommended for routine commissioning.^76^

###  Italy – Fund for Innovative Oncological and Non-oncological Medicines

 In 2017, the government launched the “Fund for Innovative Oncological and Non-oncological medicines” to solve the problem of access to innovative medicine. This fund aims (1) to facilitate early access to innovative oncology and non-oncology drugs and (2) to ensure equal access to cancer drugs for all Italians as each region administers its benefits package.^60^

 Agenzia Italiana del farmaco (AIFA) is responsible for designating whether the new active substances are deemed innovative, conditionally innovative, or not innovative. AIFA makes the decision based on three main criteria: (1) Unmet therapeutic need: High, important, moderate, scarce, and absent; (2) Added value: High, important, moderate, scarce, and absent; and (3) Quality of evidence using Grading of Recommendations Assessment, Development and Evaluation GRADE method: High, moderate, low, and very low.^60^

 The Italian government allocates US$ 1136 million annually to the Fund for innovative oncological and non-oncological medicines (US$ 568 million each for cancer and non-cancer drugs). Drugs with innovative status will be subsidized for 36 months under this innovative funding, and this will be equally applied across all regions. On the other hand, reimbursement of drugs with “conditional innovative” status will be subjected to each regional administration. Drugs with “not innovative” status will not get reimbursed under the universal health insurance system named “Servizio Sanitario Nazionale” (SSN). SSN did not require any cost-sharing for medicines.^60,78^

 After reaching the innovative status, AIFA and the pharmaceutical company need to set the reimbursement price. It was reported by Prada et al that the most common price-setting strategies were hidden discounts followed by financial-based and performance-based MEAs.^79^

 Patients receiving drugs under the innovative drug fund are subjected to registration for outcome monitoring. Information from Statista revealed expenditure on innovative drugs since the special fund embarkment. It was found that the expenses were US$ 764 million in 2017 and continuously increased to US$ 1546, US$ 1965, and US$ 2279 million in 2018-2020, respectively.^80^ It is worth noting that the expenditure for innovative drugs exceeded the 1 billion Euro budget the year after the special funding was launched. The government primarily allocated a budget of US$ 1136 million for innovative drugs, while the pharmaceutical companies were responsible for rebates exceeding expenditures.

###  Comparison of Dedicated Funds for Cancer Drugs Across Identified Countries

 We compared key characteristics of dedicated funds for cancer drugs in Hong Kong, England, and Italy.^37,43,50-61^ Dedicated funds for cancer drugs in England and Italy are similar in providing early access to innovative cancer drugs while real-world data are being collected to inform future transitioning to the normal funding mechanism. Dedicated funds for cancer drugs in Hong Kong, on the other hand, are established to provide financial assistance to patients who need cancer drugs. England’s CDF provides access to only cancer drugs. Hong Kong’s SF/CCF and Italy’s Fund for Innovative Oncological and Non-oncological Medicines provide access to both cancer and non-cancer drugs.

 The process of enlisting cancer drugs to the dedicated funds in England and Italy is done as part of the normal reimbursement pathway. Responsible authorities make the decision to include cancer drugs either under the standard drug list or dedicated funds for cancer drugs. Cancer drugs considered for listing in Hong Kong’s SF/CCF are once rejected for inclusion in the standard drug list.

 The length of coverage of cancer drugs under the dedicated funds ranges from up to 24 months in England to 36 months in Italy, after which the covered cancer drugs will be re-evaluated to make a final decision to either be reimbursable drugs under the standard drugs list or non-reimbursable drugs. Cancer drugs under dedicated funds in England and Italy will be provided under patient registry programs to monitor patient outcomes and collect real-world data. Patient outcomes are not collected in Hong Kong’s SF/CCF. Cancer drugs under the dedicated funds are provided without cost sharing in England and Italy. Hong Kong SF/CCF requires patients to pay cost-sharing depending on their household income.

 Dedicated funds for cancer drugs in all countries are funded with the annual budget allocated mainly from government sources. The expenditures of dedicated funds for cancer drugs as a percentage of pharmaceutical expenditures range from 1% in England to 5% in Hong Kong. In Hong Kong, the spending of the dedicated funds for cancer drugs is well controlled, with spending within the allocated budget. Spending of the dedicated funds for cancer drugs in England and Italy is exceeding the designated budget. However, the overspending does not cost extra budget to the government. England’s and Italy’s dedicated funds for cancer drugs have a financial control mechanism to prevent budget overspending, such as a proportional rebate to all pharmaceutical companies receiving any funding from the dedicated funds in the event of an overspend in England and Italy.

###  Comparison of Cancer Drugs in Thailand, Hong Kong, England, and Italy

 Results demonstrated that 269 unique cancer drugs were identified and divided into 15 groups according to the ATC classification system.^51,53,81-90^ Protein kinase inhibitors had the highest proportion, followed by monoclonal antibodies and miscellaneous in all four countries. All 269 drug lists were reported in Table 3.

**Table 3:** ATC Classification of Cancer Drugs in Thailand, Hong Kong, England, and Italy

**ATC classification**	**Thailand**					**Hong Kong**					**England**					**Italy**				
	**Reimbursable Under Regular Benefits Package**	**Reimbursable Under Special Programs**	**Not Reimbursable**	**Not Registered**	**Total**	**Reimbursable Under Regular Benefits Package**	**Reimbursable Under the Dedicated Drugs Fund**	**Not Reimbursable**	**Not Registered**	**Total**	**Reimbursable Under Regular Benefits Package**	**Reimbursable Under the Dedicated Drugs Fund**	**Not Reimbursable**	**Not Registered**	**Total**	**Reimbursable Under Regular Benefits Package**	**Reimbursable Under the Dedicated Drugs Fund**	**Not Reimbursable**	**Not Registered**	**Total**
Alkylating agents	8	0	2	9	19	11	1	0	7	19	11	2	0	6	19	12	0	7	0	19
Alkylating agents; platinum coordination complexes	3	0	0	0	3	3	0	0	0	3	3	0	0	0	3	3	0	0	0	3
Antibiotics, cytotoxic	6	0	2	5	13	8	1	1	3	13	6	1	0	6	13	8	0	5	0	13
Antimetabolites; antifolates	1	0	2	2	5	2	0	0	3	5	3	0	0	2	5	3	0	2	0	5
Antimetabolites; purine analogues	3	0	2	2	7	5	0	0	2	7	3	2	0	2	7	7	0	0	0	7
Antimetabolites; pyrimidine analogues	4	0	3	3	10	4	1	1	4	10	7	1	1	1	10	7	0	3	0	10
Histone deacetylase inhibitors	0	0	0	4	4	0	0	0	4	4	0	1	0	3	4	0	0	4	0	4
Hormonal agents; antiandrogens	2	2	2	2	8	3	2	1	2	8	2	3	1	2	8	7	0	1	0	8
Hormonal agents; antiestrogens (including aromatase inhibitors)	2	0	3	2	7	4	0	1	2	7	4	0	2	1	7	7	0	0	0	7
Hormonal agents; gonadotropin releasing hormone analogues	4	0	2	4	10	6	0	0	4	10	0	1	1	8	10	7	0	3	0	10
Hormonal agents; peptide hormones	1	0	1	0	2	2	0	0	0	2	2	0	0	0	2	2	0	0	0	2
Monoclonal antibodies	4	2	16	22	44	5	13	3	23	44	7	23	8	6	44	22	8	14	0	44
Protein kinase inhibitors	5	10	27	32	74	4	26	6	38	74	11	42	11	10	74	41	8	25	0	74
Topoisomerase inhibitors	3	0	0	3	6	2	0	1	3	6	3	0	0	3	6	4	0	2	0	6
Taxanes	2	0	2	0	4	2	0	1	1	4	1	2	0	1	4	3	0	1	0	4
Vinca alkaloids	3	0	1	2	6	3	0	0	3	6	3	0	0	3	6	5	0	1	0	6
Biologic response modifiers	0	0	1	3	4	1	0	1	2	4	2	0	1	1	4	2	0	2	0	4
Monoclonal antibodies and topoisomerase inhibitors	0	0	0	2	2	0	0	0	2	2	0	1	1	0	2	0	0	2	0	2
Monoclonal antibodies and cytotoxic agent	0	0	1	0	1	0	1	0	0	1	0	1	0	0	1	1	0	0	0	1
Combination	1	0	0	6	7	1	0	0	6	7	0	4	0	3	7	2	0	5	0	7
Miscellaneous	7	1	9	16	33	11	4	1	17	33	6	11	7	9	33	20	2	11	0	33
**Total**	**59**	**15**^a^	**76**	**119**	**269**	**77**	**49**^b^	**17**	**126**	**269**	**74**	**95**	**33**	**67**	**269**	**163**	**18**	**88**	**0**	**269**

Abbreviation: ATC, Anatomical Therapeutic Chemical. ^a^ Six cancer drugs in Thailand including bevacizumab, dasatinib, imatinib, nilotinib, rituximab, and trastuzumab are reimbursed under regular benefits package of Thailand with additional indications reimbursed under the OCPA. ^b^ Seven cancer drugs in Hong Kong including cetuximab, dasatinib, everolimus, imatinib, interferon alfa, rituximab, and temozolomide are reimbursed under regulars benefit package of Hong Kong with additional indications reimbursed under SF and CCF.

**Figure 2:**
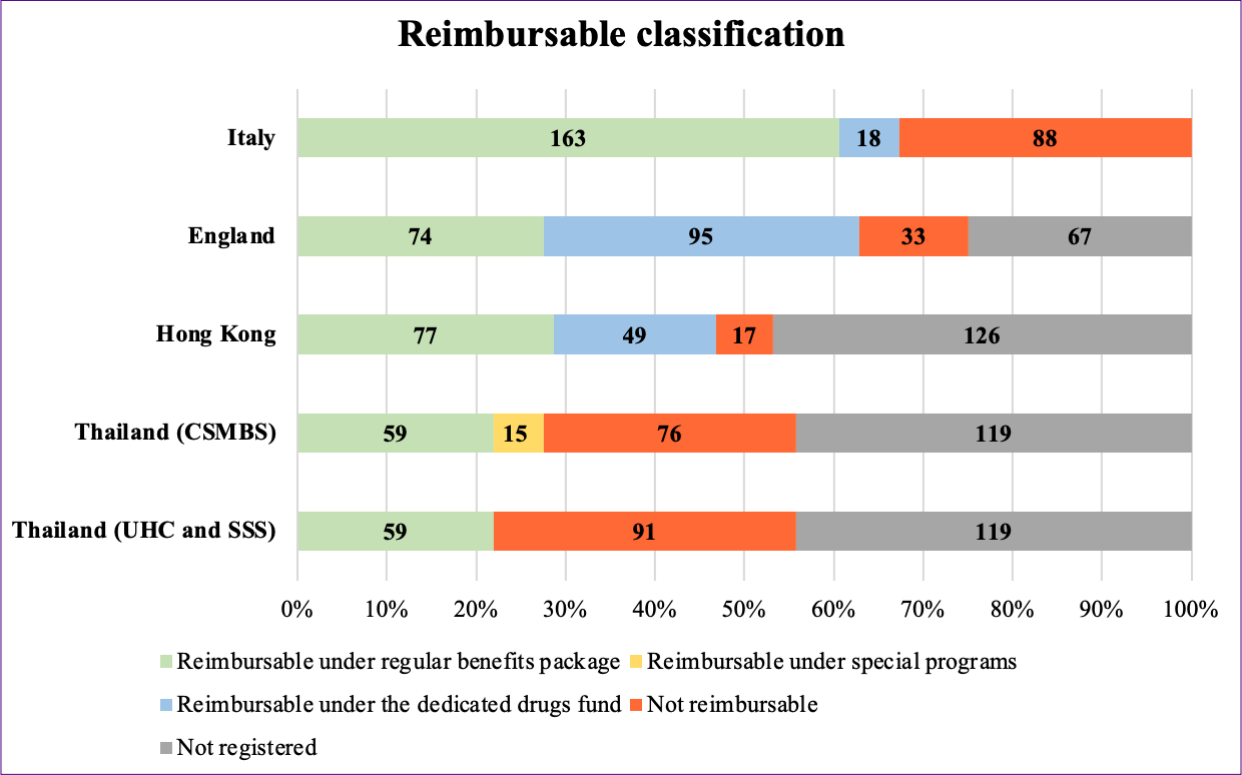
Reimbursable Classification of Cancer Drugs in Thailand, Hong Kong, England, and Italy. Abbreviations: UHC, universal health coverage; SSS, social security scheme; CSMBS, Civil Servant Medical Benefit Scheme.

 A comparison of the reimbursable classification of 269 cancer drugs across four countries was shown in Figure 2. Italy led with the highest number of cancer drugs available in the country (n = 269), followed by England (n = 202), Thailand (n = 150), and Hong Kong (n = 143). Italy also had the largest number of reimbursable cancer drugs (n = 181), followed by England (n = 169), Hong Kong (n = 126), Thailand for CSMBS (n = 74), and Thailand for UHC & SSS (n = 59). Among the reimbursable cancer drugs, Italy was the country with the highest number of cancer drugs on the regular benefits package (163 cancer drugs, 60.59%), followed by Hong Kong (77 cancer drugs, 53.84%) while England was the country with the highest proportion of drugs in the CDF (95 cancer drugs, 47.03%). Thailand not only had a lower number of reimbursements under the regular benefits package (59 cancer drugs, 39.33%) and a higher number of non-reimbursable drugs (91 cancer drugs, 60.67%) than other countries but also recorded that approximately half of all drugs were not even registered as a licensed drug.

## Discussion

 We specifically reviewed and compared the dedicated funds for cancer drugs utilized by three countries, including Hong Kong, England and Italy. Dedicated funds for cancer drugs have been established with clear objectives. These funds are typically created to enhance access to cancer drugs awaiting transition into the regular reimbursement system. The decision to facilitate access to innovative cancer drugs is distinct from other aspects of cancer care, such as cancer screening, palliative care, and end-of-life treatment, which many of these aspects are already covered by the national health insurance systems in all three countries.

 All dedicated funds are publicly financed. Two of these funds receive an annual budget, while the third is allocated a lump sum budget for a 10-year period. The annual budget allocated to the funds in England and Italy is subject to a cap, estimated to be less than 2.5% of the annual national pharmaceutical expenditure. With a fixed budget, the government can allocate funds with certainty each year and evaluate whether to continue utilizing this strategy. In contrast, the funds that receive a lump sum budget can strive for self-sustainability through prudent investment.

 The scope of dedicated drug funds may vary. Hong Kong and Italy operate the funds nationwide, while England is the only country within the United Kingdom operating the funds. Moreover, it is worth noting that in Hong Kong and Italy, dedicated funds extend their coverage to both cancer and non-cancer drugs. In contrast, dedicated funds in England specifically target funding for cancer drugs exclusively. The variation in the scope of dedicated funds may reflect the unique healthcare challenges and financial situations in each country.

 Management of the dedicated funds varies among the three countries, particularly in terms of drug listing and delisting. Two distinct pathways have emerged: parallel and sequential. In the parallel approach, a single expert committee assesses drugs for reimbursement under three options: (1) regular pharmaceutical benefits scheme, (2) dedicated funds, or (3) non-reimbursement. In contrast, the sequential pathway involves two committees. The first assesses drugs for inclusion in the regular pharmaceutical benefits scheme, with the possibility of proposing non-listed drugs for dedicated funds consideration. The parallel pathway appears more efficient than the sequential one.

 The drug selection criteria, including unmet medical need, added therapeutic value, and the quality of the evidence, are consistent across the three countries. In Italy, the quality of the evidence is of paramount importance. If the quality of the evidence is assessed as low or medium, the drugs will not qualify for full innovative status. A recent study by Jommi and Galeone reported that the innovative status is primarily influenced by the added therapeutic value and the quality of the evidence rather than the unmet need.^91^ Moreover, cancer drugs prescribed for end-of-life care should be deliberately considered with palliative care. The utilization of cancer drugs in such cases may be perceived as a signal of aggressive treatment. Aggressive treatments involving newer cancer drugs do not consistently enhance patients’ conditions or prolong the quality of life. This circumstance can potentially result in the underutilization of palliative care.^92^

 Delisting is another mechanism used in dedicated funds management. England and Italy have established fixed time frames of 24 and 36 months, respectively, for delisting drugs from dedicated funds. Once the grace period ends, the pharmaceutical companies have the option to submit the drug for evaluation under the regular pharmaceutical benefits scheme. In contrast, Hong Kong does not employ a time frame strategy but instead evaluates drugs for delisting on a case-by-case basis through the committee.

 Although dedicated funds are seen as ring-fenced, they employ various cost-control mechanisms. MEAs play a central role in cost containment in England and Italy. Financial-based MEAs are more favorable in Italy due to their operational ease and resource efficiency. In contrast, outcome-based MEAs are more favorable in England as it provides evidence for further drug selection decision.

 Cost-sharing strategies vary among these countries. In Hong Kong, tiered cost-sharing based on family income is applied within the dedicated funds, but it is not mandatory for drugs covered by the regular pharmaceutical benefits scheme. Conversely, England and Italy require cost-sharing for drugs covered by the regular pharmaceutical benefits scheme but do not impose cost-sharing within dedicated funds. Given the potentially impoverishing impact of cancer on patients and their families, the implementation of cost-sharing strategies should be approached with careful consideration.

 Proportional rebate is implemented in both England and Italy. While drug expenditures surpassing budgetary constraints have not hindered patients from continuing their treatment, they have imposed a financial burden on pharmaceutical companies. Notably, in Italy, innovative drug spending exceeded the limit during the second year of implementation, leading to pharmaceutical companies being required to make repayments. While rebates help mitigate financial risk for insurers, they concurrently place a burden on pharmaceutical companies. The dedicated funds will be seen as less attractive if the budget is inadequate and requires a large proportion of rebates.

 While dedicated drugs fund enable greater access to innovative drugs, the evaluation of their performance, including clinical outcomes and patient satisfaction, remains limited. The drugs listed in the regular pharmaceutical benefits scheme serve as a tangible trace indicating whether dedicated funds provide the opportunity for innovative drugs to prove their value.

 In 2023, an NHS report revealed that out of the 28 drugs listed under the England CDF, 24 had received recommendations from NICE for inclusion in the NHS regular reimbursement system.^93^

 When countries establish a dedicated drugs fund, it is crucial to consider both benefits and risks. While patients will experience improved drug access and an enhanced quality of life, it’s essential to acknowledge that co-pay requirements may persist in certain countries. Physicians might have access to alternative drugs better suited to their patients’ needs. However, accurately recording and reporting clinical outcomes is imperative for assessing the drugs’ potential. Payers can broaden their reimbursement drug lists to aid those in need but must judiciously manage funding sources, control budgets, and contain costs. Moreover, pharmaceutical companies, while introducing new drugs, must be willing to share risks with payers. It’s pivotal to carefully weigh these factors before implementing a dedicated drugs fund.

###  Policy Recommendations for Thailand

 Cancer is the leading cause of death in Thailand. Within the cancer care continuum, the Thai healthcare system provides limited cancer screening (Human papillomavirus screening for women aged 35 and older), cancer treatment (reimbursed cancer drugs according to established cancer protocols), and symptomatic treatment. However, cancer treatment is widely recognized as the most critical aspect. Out-of-pocket payments for drugs not listed in the NLEM are deemed unacceptable for eligible cancer patients covered by the three public health insurance systems.

 The issue of access to cancer drugs in Thailand is widely recognized, and numerous stakeholders are continuously working to address it.^6,7^ Many attempts have recently been pushed forward. In 2022, the Thai Society of Clinical Oncology proposed an updated cancer protocol. If approved by payers, these revised protocols will serve as a framework for cancer drug reimbursement across public health insurance programs in Thailand. Additionally, the sub-committee of the NLEM has recently agreed to exempt rare disease drugs from the regular HTA pathway if they are deemed lifesaving, recommended by national or international clinical practice guidelines, and fall within a reasonable budget. In the same year, the National Health Security Office (NHSO) established a new committee comprising healthcare providers, academia, representatives from the NLEM’s subcommittee, and pharmaceutical companies to address the issue of access to high-cost drugs. This new committee identified MEAs and dedicated drugs fund as attractive and feasible strategies to implement in Thailand’s healthcare system.^94^

 The proposed dedicated drugs fund should aim to enhance access to non-reimbursable drugs with proven added benefits over those in the NLEM, supported by robust evidence of their significant clinical impact. The dedicated drugs fund not only provides additional funding to the healthcare system but also establishes a maximum drug expenditure limit, ensuring that the government does not face a financial crisis while patients receive continuous treatment. The dedicated funds should encompass both cancer and non-cancer drugs, as well as rare diseases. It is imperative that all Thai citizens have equal access to drugs listed in the dedicated drugs fund, irrespective of their health insurance schemes.

 The primary source of funding to support the dedicated drugs fund should originate from the government. It is imperative for the government to commit to financing the dedicated drugs fund. An issue that warrants further discussion is the allocation of the budget to the fund. Allocating additional government tax revenue to the fund necessitates the country to make trade-offs with other meaningful projects and activities. Seeking non-governmental tax support, especially from those who will benefit from the fund or private entities seeking tax exemptions, may serve as alternative financial sources that can help ensure the sustainability of the dedicated funds. Additionally, considering other financial sources, such as revenue generated from endowment funds and donations, is also prudent.

 Regarding management, the dedicated drugs fund must establish clear drug listing and de-listing criteria, accompanied by a reasonable de-listing timeline. These criteria should be meticulously drafted, undergo a hearing process, and be pilot-tested. The drug selection process should align with the NLEM. This is particularly important as Thailand has a limited number of HTA experts. Utilizing the same expert committee is more efficient, as they can determine whether a new drug should be reimbursed under the NLEM, the dedicated funds, or not reimbursed at all.

 The fund administration should be entrusted to an existing organization, such as one of the three public health insurers. The NHSO is considered a potential candidate, as it covers over 70% of the Thai population and is regarded as the most advanced claim administrative system.

 Cost-sharing is a contentious issue in the context of Thailand. Given the country’s national-politics-driven health insurance system over the past 20 years, which promised free healthcare access to all Thai citizens, cost-sharing is perceived as the least acceptable strategy. Proposing that individuals contribute to the fund for future utilization may be a more possible approach. Additionally, establishing financial risk-sharing mechanisms between the government and pharmaceutical companies is a crucial aspect of the dedicated drugs fund. When the annual budget is exceeded, proportional rebates should also be considered.

 Information technology infrastructure is crucial for successfully implementing dedicated funds. A robust Information technology system should facilitate the tracking of drug utilization under the dedicated drugs fund, encompassing both expenditure and clinical outcomes. Furthermore, the evaluation of outcomes stemming from implementing the dedicated drugs fund should be clearly defined. This evaluation should not only encompass clinical and financial outcomes but also comprehensively assess the impact on the overall healthcare system.

 Although the concept of the dedicated drugs fund receives support from many stakeholders, it is worthwhile to consider the opportunity cost associated with its implementation when compared to investing in health education initiatives aimed at raising awareness of cancer self-examination, population-based cancer screening, and palliative care. These areas are either not well-established or are not currently covered under the public health insurance system in Thailand.

###  Limitations

 Our study has some unavoidable limitations. Firstly, the inherent nature of health policy research involving grey literature searches might lead to the omission of recent information. Future studies should consider updating data in countries where dedicated drugs fund is already in use and in countries where these funds have been newly established.

 Secondly, the classification of certain cancer drugs as reimbursable could not be thoroughly examined in England due to the unavailability of a positive drugs list, with only the negative drugs list accessible. Consequently, the absence of a positive drugs list made it challenging to determine which drugs were covered by national health insurance. Nonetheless, our findings could serve as a proxy for evaluating access to high-cost innovative cancer drugs in the studied countries. Future studies should consider conducting surveys with key informants in each country to investigate the reimbursement classification of cancer drugs.

 Thirdly, this study focused solely on access to cancer drugs in terms of reimbursement status. It did not extend its scope to the timeline for including cancer drugs in the regular benefits package and dedicated drugs fund. Consequently, we were unable to assess the efficiency of the dedicated drugs fund in terms of ensuring timely access to cancer drugs. This issue should be considered in future research.

## Conclusion

 The dedicated drugs fund is considered an attractive and feasible strategy to enhance access to non-reimbursable high-cost drugs in Thailand. Robust criteria and evaluation processes for drug inclusion in the dedicated drugs fund, as well as for transitioning drugs back into the standard reimbursement drugs list, are crucial. This ensures efficient management and sustained patient access to needed medicines post-exit. Insights from other countries offer a promising solution for limited medication access. To implement the dedicated drugs fund, the responsible organization must thoroughly prepare its structures, objectives, operational plan, funding sources, and management system. Health insurers must balance providing additional cancer treatments and benefits for overall member well-being.

## Acknowledgments

 The authors would like to thank Mr. Krit Yodinlom for his diligent support in English language proofreading of this work.

## Ethical issues

 Not applicable.

## Competing interests

 Authors declare that they have no competing interests.

## Funding

 This work was supported by MSD (Thailand) Ltd. The funder had no role in any part of the work, including the design and conduct of the study, as well as manuscript preparation.

## Supplementary files

Supplementary file 1 contains Table S1.

Supplementary file 2 contains Table S2.
