# Supplementary material for: Scoping Review of International Experience of a Dedicated Fund to Support Patient Access to Cancer Drugs: Policy Implications for Thailand
Source: Int J Health Policy Manag. 2024 Jan 28;13:7768. doi: 10.34172/ijhpm.2023.7768 (PMC11607595; doi:10.34172/ijhpm.2023.7768)
Supplement: Supplementary file 2 — contains Table S2. [file ijhpm-13-7768-s002.pdf]

Article title: Scoping Review of International Experience of a Dedicated Fund to Support Patient Access to Cancer Drugs: Policy Implications for Thailand

Journal name: International Journal of Health Policy and Management (IJHPM)

Authors' information: Parnnaphat Luksameesate, Osot Nerapusee, Chanthawat Patikorn, Puree Anantachoti*

Department of Social and Administrative Pharmacy, Faculty of Pharmaceutical Sciences, Chulalongkorn University, Bangkok, Thailand.

***Correspondence to:** Puree Anantachoti; Email: puree.a@pharm.chula.ac.th

Citation: Luksameesate P, Nerapusee O, Patikorn C, Anantachoti P. Scoping review of international experience of a dedicated fund to support patient access to cancer drugs: policy implications for Thailand. Int J Health Policy Manag. 2024;13:7768. doi:10.34172/ijhpm.2023.7768

Supplementary file 2

Table S2 Search strategies

**Database**	**Date**	**Search term**	**Results**
PubMed	May 12, 2021	((("cancers"[All Fields] OR "cancerated"[All Fields] OR "canceration"[All Fields] OR "cancerization"[All Fields] OR "cancerized"[All Fields] OR "cancerous"[All Fields] OR "neoplasms"[MeSH Terms] OR "neoplasms"[All Fields] OR "cancer"[All Fields] OR "cancers"[All Fields]) AND "drug"[All Fields]) OR "antineoplastic agents"[MeSH Terms]) AND ("financial management"[MeSH Terms] OR ("financial"[All Fields] AND "management"[All Fields]) OR "financial management"[All Fields] OR "fund"[All Fields])<br>Filters: Humans, from 2010 - 3000/12/12	2834
Embase	May 12, 2021	(((‘cancers’ OR ‘cancerated’ OR ‘canceration’ OR ‘cancerization’ OR ‘cancerized’ OR ‘cancerous’ OR ‘neoplasms’ ‘cancer’) AND ‘drug’) OR ‘antineoplastic agents’) AND (‘financial management’] OR (‘financial’ AND ‘management’ OR ‘fund’)<br>Filter: From 2010	2019
